# Systematic Reviews Comparing Direct and Indirect Restorations: An Umbrella Review That Examines Restoration Type and Confidence in Results

**DOI:** 10.1002/cre2.70149

**Published:** 2025-05-26

**Authors:** Mona Kimmel, Clovis Mariano Faggion

**Affiliations:** ^1^ Private Practice Ladbergen NRW Germany; ^2^ Department of Periodontology and Operative Dentistry, Faculty of Dentistry University Hospital Münster Münster Germany

**Keywords:** dental restoration, direct restoration, indirect restoration, methodological quality, overview of systematic reviews, restoration failure

## Abstract

**Objectives:**

There are two technologies for restoring individual structurally defective teeth. A direct restoration is applied chairside in one appointment, while an indirect restoration needs to be lab‐produced before application. This umbrella review of previous English systematic reviews was conducted to determine if there was any difference between the two restoration types in adults regarding failure and the review methodological quality.

**Materials and Methods:**

On November 7, 2023, three databases (PubMed, Web of Science, and Cochrane) were searched for systematic reviews comparing direct and indirect restorations. Two researchers independently selected the studies and extracted the information from the full texts of the articles. The methodological quality of the reviews was assessed with the measurement tool to assess systematic reviews (AMSTAR‐2) checklist.

**Results:**

Initially, 436 articles were identified; after screening, a total of 20 systematic reviews were included. Overall, the reviews indicated that both restorations produced similar results. There was a preference for direct restoration of small defects and indirect restoration for teeth with fewer than two remaining walls. Direct restoration was more cost and production‐efficient, but indirect restoration offered better long‐term results for larger defects. The overall confidence in the systematic review results ranged from critically low to high.

**Conclusions:**

Either restoration is a valid option for restoring damaged teeth. The success of the restoration depends on the patient, operator, remaining tooth structure, and restoration material. Because of the high heterogeneity of patients, teeth, and material factors, there is no recommendation on the restoration type.

## 1 Introduction

Dental restorations are produced to replace lost tooth tissue that appears from carious or non‐carious lesions (Chadwick et al. 2001), such as tooth wear (Muts et al. 2014; Ahmed and Murbay 2016; Mesko et al. 2016; Hardan et al. 2022), developmental disorders (Strauch and Hahnel 2018), and cusp fracture (Chadwick et al. 2001; Angeletaki et al. 2016; da Veiga et al. 2016). There are two types of restoration to replace missing tooth structure: direct and indirect restoration (Downer et al. 1999; Chadwick et al. 2001; Grivas et al. 2014; Muts et al. 2014; Ahmed and Murbay 2016; Sequeira‐Byron et al. 2015; van de Sande et al. 2016; Mesko et al. 2016; Afrashtehfar, Ahmadi, et al. 2017; Angeletaki et al. 2016; Afrashtehfar, Emami, et al. 2017; da Veiga et al. 2016; Suksaphar et al. 2017; Strauch and Hahnel 2018; Azeem and Sureshbabu 2018; Vetromilla et al. 2020; de Kuijper et al. 2023; McGrath and Bonsor 2022; Hardan et al. 2022; Kamposiora et al. 2023). Direct restoration is mainly indicated for small cavities or defects with less structural loss. Therefore, the operator typically requires only one session to apply plastic filling material directly to the preparation. Since the EU‐wide amalgam ban (Minamata Convention), common materials used are resin‐based composites and glass ionomer cements.

Glass ionomer cements have been used more rarely in recent years (Downer et al. 1999; Chadwick et al. 2001; Strauch and Hahnel 2018; Vetromilla et al. 2020; de Kuijper et al. 2023). Because of its low oral durability, glass ionomer was mainly indicated for defects in primary teeth (Chadwick et al. 2001) or is suitable as an intermediate material (Vetromilla et al. 2020). It is now becoming increasingly important for the filling therapy of Class I, II, and V cavities (G.V. Black) in adults. Based on aesthetics and adaptation, amalgam was indicated for posterior teeth (Downer et al. 1999; Chadwick et al. 2001; van de Sande et al. 2016; Afrashtehfar, Ahmadi, et al. 2017; Afrashtehfar, Emami, et al. 2017; Strauch and Hahnel 2018; Vetromilla et al. 2020), but its major advantage was a fast and uncomplicated application.

Composites can be used for anterior and posterior teeth (Hardan et al. 2022; Kamposiora et al. 2023). The advantages of direct composite restorations regarding failure are their mechanical properties, which make them repairable, modifiable, and able to absorb forces (Grivas et al. 2014). Disadvantages include polymerization shrinkage and lower strength (Grivas et al. 2014).

Indirect restoration is indicated for larger defects (Downer et al. 1999; Vetromilla et al. 2020). Before the restoration can be adapted, the operator needs to make an impression of the prepared tooth. Based on the impression, a restoration is fabricated in a laboratory. This means that at least one more session is needed before the restoration can be placed. Furthermore, it is possible to create a 3D Scan of a model or directly in the patient's mouth. Based on this scan, the restoration can be digitally constructed. The data set is then sent to a milling machine or a printer, which produces the restoration.

Materials available for indirect restorations are cast alloys, ceramics, and UDMA‐based materials (Chadwick et al. 2001; Grivas et al. 2014; Muts et al. 2014; Mesko et al. 2016; Kamposiora et al. 2023). The advantages of indirect restoration regarding failure are superior wear resistance and strength (Grivas et al. 2014). While soft metals, especially gold, adjust to the situation in the mouth and do not possess excessive strength, ceramics are nonmalleable and do not offer this advantage. Although several studies have reported that direct and indirect restorations provide comparable results (Downer et al. 1999; Chadwick et al. 2001; Grivas et al. 2014; Muts et al. 2014; Ahmed and Murbay 2016; Mesko et al. 2016; Angeletaki et al. 2016; Afrashtehfar, Emami, et al. 2017; da Veiga et al. 2016; Suksaphar et al. 2017; Azeem and Sureshbabu 2018; de Kuijper et al. 2023; McGrath and Bonsor 2022; Hardan et al. 2022; Kamposiora et al. 2023) it is important to understand which approach can be considered the first‐line treatment to repair lost tooth tissue.

Systematic reviews are used to compile evidence on treatments for a specific condition. When conducted well, these reviews provide the best available sources for the development of clinical guidelines (Morrison et al. 2012). Therefore, their methodological quality assessment is a sine qua non for their adequate appraisal.

This overview (umbrella review) of systematic reviews comparing direct and indirect restorations had two objectives: (1) to systematically assess the differences in restoration failure between direct and indirect restorations and (2) to assess the methodological quality of systematic reviews comparing direct and indirect restorations with a validated checklist.

## 2 Materials and Methods

### 2.1 Research Questions

This umbrella review of systematic reviews was developed to answer two research questions: (1) Is there any difference between direct and indirect restorations regarding restoration failure? (2) What is the methodological quality of systematic reviews focusing on direct and indirect dental restorations? Therefore, the overview followed a patient–intervention–comparison–outcome (PICO) framework:

P → Adults

I → Direct restorations

C → Indirect restorations

O → Restoration failure

### 2.2 Eligibility Criteria

Systematic reviews of adult human participants focusing on direct and indirect restorations were included in the present study. Therefore, only systematic reviews that included both direct and indirect restorations were included. Other types of reviews, such as narrative reviews, were excluded. We considered a review “systematic” when the authors explicitly reported their intention to conduct a “systematic review.” Systematic reviews of non‐interventional studies, such as those that investigated tools or approaches to detect caries around restorations or crowns, were excluded. In addition, only systematic reviews published in English were included.

### 2.3 Definition of Direct and Indirect Restorations and Outcome Measures

Direct restorations were defined as those directly applied to the patient's chairside using plastic filling material. Indirect restorations were defined as those that required production in a laboratory or were produced outside the mouth of the patient with the use of extra equipment, more specifically, inlays, overlays, partial crowns, and crowns. The outcome measures used to reflect restoration failure in the present study were tooth/restoration fracture, marginal integrity, aesthetics, endodontic problems, and any measure of efficiency.

### 2.4 Search Strategy

Three major electronic databases (PubMed, Web of Science, and Cochrane) were searched for systematic reviews using specific search strategies tailored to each. The searches were performed on November 7, 2023, and included publications from database inception up to November 7, 2023. The detailed search strategies used in each database are reported in the supplementary file. Furthermore, the reference lists of the systematic reviews initially retrieved from the databases were scrutinized for further reviews.

### 2.5 Data Selection

The titles/abstracts of the documents were first assessed for inclusion. If they indicated that the documents did not meet the eligibility criteria, the documents were excluded, and the reasons for exclusion were recorded. The full texts of documents initially selected by title/abstract were then assessed, and if the text indicated that a document did not meet the eligibility criteria, it was excluded, and the reason for exclusion was recorded. Two assessors selected a sample of eligible studies to compare results and achieved good agreement (at least 80%); the remainder were selected by one reviewer (Morrison et al. 2012).

### 2.6 Data Extraction

The following information was extracted from the selected articles: (a) title; (b) journal name; (c) impact factor (IF); (d) number of citations; (f) systematic review with or without meta‐analysis (yes/no); (g) systematic review registered (yes/no); (h) country/continent of the first and last authors; (i) number of authors; (j) conflict of interest reported (yes/no); (k) sponsorship reported (yes/no); (l) type of primary studies included in the systematic review; (m) type of interventions; and (n) type of outcomes. Two assessors extracted the information from a sample of eligible studies to compare results and achieved good agreement (at least 80 percent), with the remainder extracted by one reviewer (Shea et al. 2017).

### 2.7 Methodological Assessment of Systematic Reviews

The methodological quality of the systematic reviews was assessed with the measurement tool to assess systematic reviews (AMSTAR‐2) checklist (Shea et al. 2017). The checklist contains 16 items relating to the different domains of a systematic review. AMSTAR‐2 has been validated (Shea et al. 2017). Items were assessed and rated at three different levels: “Yes” (when the item was adequately reported), “Partial Yes” (when the item was partially reported), and “No” (when the item was not reported). If the item was not relevant (e.g., items related to a meta‐analysis when assessing a systematic review without meta‐analysis), the item was rated as “NA” (not applicable).

We also determined the overall confidence in the results of the systematic reviews (Shea et al. 2017). This assessment was conducted by assessing critical domains of the reviews (Items 2, 4, 7, 9, 11, 13, and 15) and noncritical (Items 1, 3, 5, 6, 8, 10, 12, 14, and 16). The overall confidence was rated on four different levels: high, moderate, low, and critical low. In the present umbrella review, to calculate overall confidence, “Partial Yes” had the same rating as “Yes.” The criteria for determining the levels of confidence were weakness in the domains that could be critical: high (no or noncritical weakness), moderate (more than one noncritical weakness), low (one critical flaw with or without noncritical weaknesses), and critically low (more than one critical flaw with or without noncritical weaknesses).

## 3 Results

### 3.1 Systematic Reviews Included

**Figure 1:**
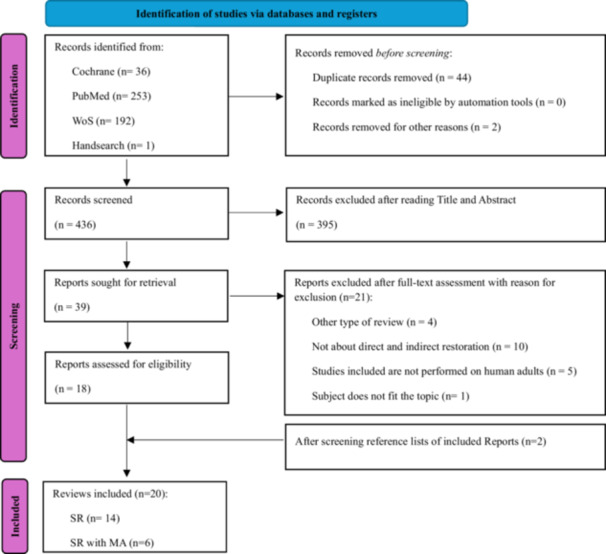
PRISMA Flowchart.

A total of 484 records from three different databases (PubMed, Cochrane Library, and Web of Science) were identified as relevant, as screened from the electronic searches (Table S1). After the exclusion of duplicates, 436 records were screened and assessed for eligibility. A total of 395 records were excluded after reading the title and abstract; the other 21 records were discarded after reading the full text (Table S2). Eighteen records met all eligibility criteria. Two systematic reviews were added after searching the reference lists of the included full texts. The selection process is reported in Figure 1 (preferred reporting items for systematic reviews and meta‐analyses [PRISMA] flowchart).

### 3.2 Characteristics of Systematic Reviews

The articles included were published between 1999 and 2023. Seven systematic reviews conducted a meta‐analysis (Chadwick et al. 2001; Angeletaki et al. 2016; Afrashtehfar, Emami, et al. 2017; da Veiga et al. 2016; Vetromilla et al. 2020; de Kuijper et al. 2023; Kamposiora et al. 2023). Ten of the included systematic reviews were from Europe (Downer et al. 1999; Chadwick et al. 2001; Grivas et al. 2014; Muts et al. 2014; Sequeira‐Byron et al. 2015; Angeletaki et al. 2016; Strauch and Hahnel 2018; de Kuijper et al. 2023; McGrath and Bonsor 2022; Kamposiora et al. 2023), four were from Asia (Ahmed and Murbay 2016; Suksaphar et al. 2017; Azeem and Sureshbabu 2018; Hardan et al. 2022), two were from North America (Afrashtehfar, Ahmadi, et al. 2017; Afrashtehfar, Emami, et al. 2017), and four were from South America (van de Sande et al. 2016; Mesko et al. 2016; da Veiga et al. 2016; Vetromilla et al. 2020). A total of 513 primary studies were included in the systematic review. This included prospective and retrospective non‐randomized studies, as well as randomized controlled trials (RCTs). The total number of patients in the studies included in the systematic reviews varied from 15 to 259,123 (median 623). Half of the systematic reviews reported data on restoration in posterior single vital teeth (Downer et al. 1999; Chadwick et al. 2001; Grivas et al. 2014; van de Sande et al. 2016; Angeletaki et al. 2016; Afrashtehfar, Emami, et al. 2017; da Veiga et al. 2016; Azeem and Sureshbabu 2018; Vetromilla et al. 2020; McGrath and Bonsor 2022). Only two systematic reviews examined restorations in anterior and posterior single vital teeth (Hardan et al. 2022; Kamposiora et al. 2023). Four systematic reviews were restorations on endodontically treated teeth (Sequeira‐Byron et al. 2015; Afrashtehfar, Ahmadi, et al. 2017; Suksaphar et al. 2017; de Kuijper et al. 2023), three were on worn teeth (Muts et al. 2014; Ahmed and Murbay 2016; Mesko et al. 2016), and one concentrated on patients with amelogenesis imperfecta (Strauch and Hahnel 2018).

Systematic reviews on direct restorations focused on Class I and II adhesive composites, amalgam, glass ionomer, fillings with posts for endodontically treated teeth, and build‐ups for worn teeth. Systematic reviews on indirect restorations focused on composite, metal, and ceramic crowns; inlays and onlays; post and core crowns for endodontically treated teeth; and full‐coverage as well as partial‐coverage crowns for worn teeth. There were four main outcomes reported: restoration performance, survival, longevity, and failure. Detailed information on the included systematic reviews is presented in Tables 1, 2, 3.

**Table 1:** Characteristics of the systematic reviews.

References	Review Design	Journal	IF	Number of databases	Study Type	Sponsorship	COI
Downer et al. (1999)	SR	*British Dental Journal*	2.0	5	Retro., prosp., NRS	N	N
Chadwick et al. (2001)	SR + MA	*NHS Centre for Reviews and Dissemination*		16	Retro., prosp.	Y	N
Grivas et al. (2014)	SR	*European Journal of Prosthodontics and Restorative Dentistry*	1.1	3	RCT, CCT	N	N
Muts et al. (2014)	SR	*Journal of Prosthetic Dentistry*	4.3	2	Case report, case series	N	N
Ahmed and Murbay (2016)	SR	*Journal of Prosthetic Dentistry*	4.3	2	Prosp., retrosp.	Y	Y
Sequeira‐Byron et al. (2015)	SR	*Cochrane Database of Systematic Reviews*	8.8	6	RCT	Y	Y
van de Sande et al. (2016)	SR	*Operative Dentistry*	1.4	3	RCT, NRSI (cohort, historical cohort), prosp.	Y	Y
Mesko et al. (2016)	SR	*Journal of Dentistry*	4.8	1	Prosp., retrosp., RCT, NRS	N	Y
Afrashtehfar, Ahmadi, et al. (2017)	SR	*International Endodontic Journal*	5.4	3	RCT, retrosp.	Y	Y
Angeletaki et al. (2016)	SR + MA	*Journal of Dentistry*	4.8	4	RCT	Y	Y
Afrashtehfar, Emami, et al. (2017)	SR	*Journal of Prosthetic Dentistry*	4.3	4	RCT, observational studies	Y	N
da Veiga et al. (2016)	SR + MA	*Journal of Dentistry*	4.8	8	RCT	Y	Y
Suksaphar et al. (2017)	SR	*Restorative Dentistry & Endodontics*	—	5	Retrosp. cohort study, prosp. cohort study, RCT	Y	Y
Strauch and Hahnel (2018)	SR	*Journal of Prosthodontics*	3.4	3	RCT, prosp., retrosp. clinical studies	N	Y
Azeem and Sureshbabu (2018)	SR	*Journal of Conservative Dentistry*	—	3	RCT, CCT	Y	Y
Vetromilla et al. (2020)	SR	*Journal of the American Dental Association*	3.1	4	RCT, prosp. and restrosp. NRS	Y	Y
de Kuijper et al. (2023)	SR + MA	*Journal of Prosthetic Dentistry*	4.3	3	RCT, prosp. and retrsosp. Clinical studies	Y	Y
McGrath and Bonsor (2022)	SR	*British Dental Journal*	2.0	5	RCT, prosp. and retrosp. NRS	Y	Y
Hardan et al. (2022)	SR	*MDPI Bioengineering*	3.8	5	RCT, clinical study, observational study, prosp. trial, retrosp. study	Y	Y
Kamposiora et al. (2023)	SR + MA	*European Journal of Prosthodontics and Restorative Dentistry*	0.58	3	RCT, prosp., retrosp.	N	Y

Abbreviations: COI, conflict of interest; MA, meta‐analysis; N, No; NRS, not randomized controlled study; prosp, prospective study; RCT, randomized controlled trial; retrosp, retrospective study; SR, systematic review; Y, Yes.

**Table 2:** Characteristics of restorations.

References	Direct restoration	Indirect restoration
Downer et al. (1999)	Amalgam, composite resin, glass ionomer/Class I and II/posterior	Gold/inlays, onlays/posterior
Chadwick et al. (2001)	Amalgam, composite, compomer, glass ionomer (primary teeth), silicate cement (primary teeth)	Ceramic, composite, gold, metal ceramic, gallium/inlays
Grivas et al. (2014)	Composite resin	Composite, gold, ceramic
Muts et al. (2014)	Composite/interim stage/worn teeth	Composite, glass ceramic, metal, gold, metal ceramic, metal resin, zirconia ceramic/worn teeth
Ahmed and Murbay (2016)	Composite/worn teeth/anterior	Composite/worn teeth/anterior
Sequeira‐Byron et al. (2015)	Composite with post/endodontically treated teeth	Composite restorations with post, metal‐ceramic crowns/endodontically treated teeth
van de Sande et al. (2016)	Amalgam, composite/posterior	composite, ceramic/Class I and II inlays, onlays, overlays, partial crowns/posterior
Mesko et al. (2016)	Composite/build‐up (Dahl principle)	Composite (partial build‐ups, Dahl principle), ceramic, cast metal, ceramo‐metal, composite, or feldspathic ceramic/worn teeth
Afrashtehfar, Ahmadi, et al. (2017)	Amalgam, composite/intracoronal, post‐retained/root‐filled posterior teeth	Metal, ceramic, metal‐ceramic/postretained crowns, post‐free crowns/root‐filled posterior teeth
Angeletaki et al. (2016)	Composite/inlay, onlay/posterior	Composite/inlays, onlays/posterior
Afrashtehfar, Emami, et al. (2017)	Composite, amalgam/posterior	Crowns/posterior
da Veiga et al. (2016)	Composite/posterior	Composite/posterior
Suksaphar et al. (2017)	Composite/endodontically treated teeth/posterior	Full‐coverage crowns/endodontically treated teeth/posterior
Strauch and Hahnel (2018)	Composite, amalgam, glass ionomer/patients with amelogenesis imperfecta	Stainless steel crowns, resin‐based composite restorations/patients with amelogenesis imperfecta
Azeem and Sureshbabu (2018)	Composite/incremental/posterior	Composite/inlays/posterior
Vetromilla et al. (2020)	Amalgam, composite, glass ionomer, sandwich composite/large defects/posterior	Gold and metal ceramic crowns, feldspathic ceramic, glass ceramic,/large defects/posterior
de Kuijper et al. (2021)	Composite with carbon fiber posts or glass fiber posts/endodontically treated teeth/posterior	Titan, cast post and cores, metal posts, fabricated metal posts/endodontically treated teeth/posterior
McGrath and Bonsor (2022)	Composite/onlays/posterior	Tooth‐colored adhesive onlays/posterior
Hardan et al. (2022)	Composite/worn teeth/anterior and posterior	Ceramo‐metal and full gold crowns, lithium disilicate ceramic, zirconia, polymer infiltrated ceramic networks, resin composite/worn teeth/anterior and posterior
Kamposiora et al. (2023)	Composite	Full‐coverage crowns, partial coverage restorations, ceramic, feldspathic, leucite‐reinforced ceramic, zirconia, metal ceramic, glass ceramic, alumina‐based ceramic, composite resin, leucite‐reinforced glass‐ceramic, lithium disilicate, zirconia‐reinforced lithium silicate

**Table 3:** Results between direct and indirect restorations.

References	Outcome measure	MA (Yes/No)	Results/Conclusions
Downer et al. (1999)	Median survival time, survival/failure rate	No	50% of all restorations can be expected to survive between 10 and 20 years, influenced by type, material, patient, operator, practice environment, and care system. Direct and indirect restorations give comparable results.
Chadwick et al. (2001)	Time until replacement, time until failure,	—	In permanent teeth, amalgam is the most durable material, followed by inlays, composites, and glass ionomer cements.
Grivas et al. (2014)	Clinical effectiveness	No	There is insufficient evidence whether composite inlays, ceramic inlays, gold inlays, or direct composite fillings have a better longevity and better aesthetic quality but they show an equal performance regarding postoperative sensitivity. There is no recommendation for the use of direct or indirect inlays.
Muts et al. (2014)	Similarities	No	Composite resin and glass ceramic give comparable results for the final restoration of worn teeth. Both are indicated materials for the treatment of tooth wear.
Ahmed and Murbay (2016)	Survival rate	No	The study results show a 90% survival rate at 2 1/2 years, over 50% at 5 years and limited long‐term evidence for direct and indirect anterior composite restorations in tooth wear patients. Both restoration types are a viable option as a short‐term to midterm treatment modality.
Sequeira‐Byron et al. (2015)	Failure, patient‐related factors	No	There is insufficient evidence to determine whether direct or indirect restoration has better effects on root‐filled teeth. Clinicians should make their decisions based on their own clinical experience.
van de Sande et al. (2016)	Restoration survival, annual failure rate	No	Several patient factors influence the failure of restorations, regardless of the material type. Clinical decision‐making on restoration survival should include patient factors.
Mesko et al. (2016)	Failure rate	No	Most studies included presented good results for direct and indirect restorations on teeth with severe wear. Both restoration types may be a feasible option for severely worn teeth.
Afrashtehfar, Ahmadi, et al. (2017)	Failure rate	No	Endodontically treated teeth with less substance loss have a better prognosis than teeth with greater substance loss. Indirect restoration is superior to direct restoration in endodontically treated teeth.
Angeletaki et al. (2016)	Restoration failure	Yes	There was no statistically significant difference in the risk of failure between direct and indirect composite inlays and onlays. There is insufficient evidence to recommend one of the two restoration types.
Afrashtehfar, Emami, et al. (2017)	Failure rate	No	In terms of direct restoration on vital teeth, amalgam gave better results than composites, but in cases of teeth with fewer than two remaining walls, crowns gave the best results. There is insufficient evidence to suggest that direct or indirect restoration of vital teeth is preferred.
da Veiga et al. (2016)	Failure rate	Yes	There was no statistically significant difference in longevity for direct and indirect restorations. Since both restoration types provide comparable results, direct restorations should be preferred in situations that require less effort and lower costs.
Suksaphar et al. (2017)	Survival rate against fracture	No	The survival rates against fracture in endodontically treated posterior teeth with direct resin composite restorations or indirect restorations in the form of crowns were high. There was no significant difference in survival rate if loss of tooth structure was minimal to moderate.
Strauch and Hahnel (2018)	Predictability,longevity	No	In patients with amelogenesis imperfecta, indirect restorations gave better results in term of predictability and longevity than direct restorations. Patients with amelogenesis imperfecta should be treated with indirect restorations.
Azeem and Sureshbabu (2018)	Survival rate	No	There was no significant difference in the clinical performance of direct and indirect composite restorations in posterior teeth. Further research with long‐term follow‐up is needed to give concrete evidence.
Vetromilla et al. (2020)	Restoration survival, failure	Yes	Indirect restoration performs better in teeth with extensive damage, and for direct restoration, resin composites and amalgam performed better than glass ionomer and sandwich composite restorations.
de Kuijper et al. (2023)	Tooth and restoration survival	Yes	Low‐quality evidence suggested no difference between direct and indirect restorations over the short‐term survival of endodontically treated posterior teeth. Studies that include other baseline characteristics are needed to assess the influence of restoration type on tooth survival.
McGrath and Bonsor (2022)	Survival, failure mode	No	Direct and indirect composite restorations showed a time‐dependent deterioration. Both restoration types offer acceptable medium‐term survival in posterior teeth.
Hardan et al. (2022)	Clinical performance	No	The discoveries concerning the performance of restoration types for treating tooth wear were contradictory. There is no evidence for a higher clinical performance of one technique.
Kamposiora et al. (2023)	Failure rate	Yes	Direct and indirect restorations in single teeth showed no significant difference for the annual failure rates. There was moderate quality of evidence but due to the high heterogeneity there is no recommendation for one restoration type.

### 3.3 Direct and Indirect Restorations

Three different materials were applied for direct restoration: amalgam, composites, and glass ionomer. Six systematic reviews included studies on amalgam (Downer et al. 1999; Chadwick et al. 2001; van de Sande et al. 2016; Afrashtehfar, Ahmadi et al. 2017; Strauch and Hahnel 2018; Vetromilla et al. 2020), and five included studies about the use of glass ionomer cement for definitive restoration of permanent teeth (Chadwick et al. 2001; Strauch and Hahnel 2018; Vetromilla et al. 2020; de Kuijper et al. 2023; Downer et al. 1999). The other systematic reviews included only studies on composite resins for direct use (Grivas et al. 2014; Muts et al. 2014; Ahmed and Murbay 2016; Sequeira‐Byron et al. 2015; Mesko et al. 2016; Angeletaki et al. 2016; Afrashtehfar, Emami, et al. 2017; da Veiga et al. 2016; Suksaphar et al. 2017; Azeem and Sureshbabu 2018; McGrath and Bonsor 2022; Hardan et al. 2022; Kamposiora et al. 2023). For indirect restorations, most systematic reviews included studies on all common restoration materials: composites, ceramics, and metals. Three reviews concentrated, especially on comparing composites as direct and indirect restoration material (Ahmed and Murbay 2016; Angeletaki et al. 2016; da Veiga et al. 2016). Regardless of the material type, restoration failure was influenced by several conditions. Longevity depends on the operator, practice environment, and type of care system (Downer et al. 1999). Other important factors for the success of direct or indirect restorations are patient factors (van de Sande et al. 2016), tooth location, tooth type, and notably the remaining tooth structure (Afrashtehfar, Ahmadi, et al. 2017; Afrashtehfar, Emami, et al. 2017; Strauch and Hahnel 2018).

Direct restorations have shown a higher success rate for teeth with minimal to moderate substance loss than for teeth with less tissue (Downer et al. 1999; Afrashtehfar, Ahmadi, et al. 2017; Afrashtehfar, Emami, et al. 2017; Azeem and Sureshbabu 2018). Afrashtehfar, Emami, et al. (2017) concluded that direct restorations were a valid option in posterior vital teeth with two or more remaining coronal walls. However, in cases with teeth that had less remaining structure, indirect restoration in the form of crowns would be preferred (Afrashtehfar, Emami, et al. 2017). Strauch and Hahnel (2018) determined that there were better results in patients with amelogenesis imperfecta when restorations were indirectly placed because the patient's enamel was qualitatively changed and did not offer a foundation for the good bonding strength of composite resins. Without considering qualitative changes of tooth substance and particularly severe substance loss, direct and indirect restoration offered acceptable medium‐term survival in posterior teeth (McGrath and Bonsor 2022) and showed no significant difference in performance in teeth with minimal to moderate substance loss (Suksaphar et al. 2017).

According to Downer et al. (1999), “50% of all restorations can be expected to survive between 10 and 20 years.” Overall, 60% of the included systematic reviews concluded that both techniques offered comparable results (Grivas et al. 2014; Muts et al. 2014; Ahmed and Murbay 2016; Angeletaki et al. 2016; McGrath and Bonsor 2022) or showed no significant difference in the results (Angeletaki et al. 2016; da Veiga et al. 2016; Suksaphar et al. 2017; Azeem and Sureshbabu 2018; de Kuijper et al. 2023; Kamposiora et al. 2023). In two of the included systematic reviews, the authors stated that there was no evidence to prefer one technique over the other (Mesko et al. 2016; Hardan et al. 2022). Vetromilla et al. (2020) concluded that direct composite and amalgam restorations performed better for the "annual failure rate", but indirect restorations showed the best "overall longevity". Regarding efficiency, six systematic reviews reported the cost‐effectiveness of direct restorations (Chadwick et al. 2001; Grivas et al. 2014; Ahmed and Murbay 2016; da Veiga et al. 2016; Vetromilla et al. 2020; Hardan et al. 2022), two added the ease of processing with this type (Grivas et al. 2014; Vetromilla et al. 2020). Vetromilla et al. (2020) acknowledged the benefit of less invasive preparation for direct composite restorations. On this basis, there was a consensus that resin composites were the preferred choice for small defects in daily practice (Vetromilla et al. 2020).

### 3.4 Methodological Quality and Overall Confidence in the Results

According to the AMSTAR‐2 checklist, most systematic reviews presented deficiencies in justification for the design of included studies (Item 3), reporting funding of included studies (Item 10), protocol for the review methods (Item 2), and listing the excluded articles (Item 7). In contrast, most of the reviews reported the PICO questions, performed study selection (Item 5) and data extraction (Item 6) in duplicate, discussed the risk of bias (Item 13), and heterogeneity in the results (Item 14) and provided a detailed description of the included studies (Item 8) (Table 4).

**Table 4:** AMSTAR 2 Results.

References	PICO (Item 1)	Review methods protocolled (Item 2)	Justification for study design (Item 3)	Search strategy (Item 4)	Performance of data inclusion (Item 5)	Performance of exclusion from including data (Item 6)	List of excluded articles (Item 7)	Detailed study description (Item 8)	RoB assessment (Item 9) (RCT/NRSI)	Funding of included studies reported (Item 10)	Data combination in a meta‐analysis (Item 11)	Impact of RoB on meta‐analysis is discussed (Item 12)	Discussion of RoB in the results (Item 13)	Discussion of heterogeneity in the results (Item 14)	Investigation of publication bias (Item 15)	Report about conflict of interest (Item 16)	Overall confidential
Downer et al. (1999)	Y	Y	Y	PY	Y	Y	PY	N	N	N	NMA	NMA	N	Y	NMA	N	Critically low
Chadwick et al. (2001)	Y	Y	Y	Y	Y	Y	Y	Y	PY	Y	NMA	NMA	Y	Y	Y	Y	High
Grivas et al. (2014)	Y	Y	N	PY	Y	Y	N	Y	PY	N	NMA	NMA	Y	Y	NMA	N	Low
Muts et al. (2014)	N	N	Y	PY	N	N	N	PY	N	N	NMA	NMA	N	N	NMA	N	Critically low
Ahmed and Murbay (2016)	N	PY	N	PY	Y	Y	Y	Y	PY	N	NMA	NMA	Y	Y	NMA	Y	Moderate
Sequeira‐Byron et al. (2015)	Y	Y	N	PY	Y	Y	Y	Y	Y	Y	NMA	NMA	Y	Y	NMA	Y	High
van de Sande et al. (2016)	Y	N	N	N	Y	Y	N	Y	PY	N	NMA	NMA	Y	N	NMA	Y	Critically low
Mesko et al. (2016)	Y	N	Y	N	Y	Y	N	PY	Y	N	NMA	NMA	Y	Y	NMA	Y	Critically low
Afrashtehfar, Ahmadi, et al. (2017)	Y	Y	N	PY	Y	Y	Y	Y	RCT: PY/NRSI: Y	Y	NMA	NMA	Y	Y	NMA	Y	High
Angeletaki et al. (2016)	Y	N	Y	PY	Y	Y	Y	Y	Y	N	Y	Y	Y	Y	N	Y	Critically low
Afrashtehfar, Emami, et al. (2017)	Y	PY	N	PY	Y	Y	Y	Y	RCT: PY/NRSI: Y	Y	Y	Y	Y	N	N	N	Low
da Veiga et al. (2016)	Y	Y	Y	PY	Y	Y	Y	Y	Y	N	Y	Y	Y	Y	N	Y	Moderate
Suksaphar et al. (2017)	Y	Y	N	PY	N	N	Y	PY	PY	N	NMA	NMA	Y	Y	NMA	Y	Moderate
Strauch and Hahnel (2018)	Y	N	Y	N	Y	Y	N	PY	N	N	NMA	NMA	N	N	NMA	Y	Critically low
Azeem and Sureshbabu (2018)	Y	N	N	PY	N	N	N	PY	RCT: PY/NRSI: N	N	NMA	NMA	N	N	NMA	Y	Critically low
Vetromilla et al. (2020)	Y	Y	Y	PY	Y	Y	Y	Y	Y	Y	Y	Y	Y	Y	N	Y	Low
de Kuijper et al. (2023)	Y	Y	N	PY	Y	Y	N	Y	Y	N	Y	Y	Y	Y	N	N	Critically low
McGrath and Bonsor (2022)	Y	PY	N	PY	Y	Y	Y	Y	Y	N	NMA	NMA	Y	N	NMA	Y	Moderate
Hardan et al. (2022)	Y	PY	N	PY	Y	Y	PY	PY	Y	N	NMA	NMA	N	N	NMA	Y	Low
Kamposiora et al. (2023)	Y	Y	N	PY	Y	Y	N	Y	Y	N	Y	Y	Y	Y	Y	N	Low

Abbreviations: N, No; NMA, no meta‐analysis; PICO, population, intervention, comparator group, outcome; RoB, risk of bias; Y, Yes;.

Among the 20 systematic reviews included, three were rated high in overall confidence according to the AMSTAR‐2 statements (Chadwick et al. 2001; Sequeira‐Byron et al. 2015; Afrashtehfar, Ahmadi, et al. 2017). Four were rated with moderate overall confidence (Ahmed and Murbay 2016; da Veiga et al. 2016; Suksaphar et al. 2017; McGrath and Bonsor 2022), five were rated with low overall confidence (Grivas et al. 2014; Afrashtehfar, Emami, et al. 2017; Vetromilla et al. 2020; Hardan et al. 2022; Kamposiora et al. 2023), and eight were rated with critically low overall confidence (Downer et al. 1999; Muts et al. 2014; van de Sande et al. 2016; Mesko et al. 2016; Angeletaki et al. 2016; Strauch and Hahnel 2018; Azeem and Sureshbabu 2018; de Kuijper et al. 2023) (Table 4).

## 4 Discussion

### 4.1 Main Findings

The present umbrella review of systematic reviews does not indicate a preference for direct or indirect restoration. Rather, the success or failure of both restoration types depends on several factors related to the patient (gender, age, caries risk, and parafunctional habits), teeth (type and structure), operator (technique and experience), and material properties (filler matrix of composites, alloy, type of ceramic, and bonding system). Most of the included systematic reviews provided low and critically low confidence in the results, as rated using the AMSTAR 2 checklist.

### 4.2 Interpretation of the Results

Most of the selected systematic reviews that were studies on single vital teeth showed comparable results for both restoration types. Through the use of a meta‐analysis, the work of Angeletaki et al. (2016), da Veiga et al. (2016), and Kamposiora et al. (2023) led to this conclusion. Afrashtehfar, Emami, et al. (2017) carried out a study that supported the influence of the remaining tooth structure. Some systematic reviews reported clinical (Mesko et al. 2016; Afrashtehfar, Ahmadi, et al. 2017; Azeem and Sureshbabu 2018; McGrath and Bonsor 2022; Hardan et al. 2022) and methodological heterogeneity (Downer et al. 1999; Grivas et al. 2014; Ahmed and Murbay 2016; Mesko et al. 2016; Afrashtehfar, Ahmadi, et al. 2017; Suksaphar et al. 2017; Azeem and Sureshbabu 2018; McGrath and Bonsor 2022; Hardan et al. 2022) which makes a statistical comparison in the form of a meta‐analysis challenging, if not impossible. Downer et al. (1999) and van de Sande et al. (2016) pointed out that more factors than material and method influence the success of a restoration in single vital teeth. There are patient‐related factors, such as gender (van de Sande et al. 2016), caries risk (da Veiga et al. 2016; Vetromilla et al. 2020), bruxism (da Veiga et al. 2016), and occlusal stress (Vetromilla et al. 2020) as well as factors dependent on the operator (Downer et al. 1999) and its experience (Ahmed and Murbay 2016 ).

Another important factor in the success or failure of restoration is the remaining tooth structure (Afrashtehfar, Emami, et al. 2017; Vetromilla et al. 2020; Suksaphar et al. 2017). There is a higher failure rate for teeth with fewer than two remaining walls, and for teeth with less structure, indirect restoration should be preferred (Afrashtehfar, Emami, et al. 2017). Vetromilla et al. (2020) summarized that the risk of failure for direct and indirect restoration increases by 30%–40% for every extra surface added. With regard to efficiency, direct restorations appear to be a better choice for small cavities. Both restoration types show comparable results in longevity (Vetromilla et al. 2020), but direct restorations bring lower costs (da Veiga et al. 2016; Vetromilla et al. 2020; McGrath and Bonsor 2022) and more time effectiveness (da Veiga et al. 2016; Vetromilla et al. 2020) due to less invasive tooth preparation and a simpler technique (Vetromilla et al. 2020) compared to indirect restorations.

In endodontically treated teeth, the restorations showed comparable results, regardless of whether they were placed directly or indirectly (Afrashtehfar, Ahmadi, et al. 2017; Suksaphar et al. 2017; de Kuijper et al. 2023). Sequeira‐Byron et al. (2015) and Afrashtehfar, Ahmadi, et al. (2017) considered that more evidence was needed. de Kuijper et al. (2023) regretted that the reviewed studies were only short‐term and only provided low‐quality evidence. For example, it was reported that for single vital teeth, the failure rate was related to the amount of remaining tooth tissue, and therefore, it was also valid for endodontically treated teeth (Afrashtehfar, Ahmadi, et al. 2017). In other words, the more tooth tissue there is, the lower the failure rate. This was supported by Suksaphar et al. (2017), who reported a restoration survival of over 90% for teeth with “minimum to moderate” structure loss and a mean follow‐up of 42–48 months.

Muts et al. (2014), Mesko et al. (2016), and Hardan et al. (2022) compared direct and indirect restoration for worn teeth. They also reported that no method demonstrated superiority over the other. Ahmed and Murbay (2016) compared only composite restorations and achieved good results for both techniques for short‐ to midterm treatments of 2,5 up to 5 years. Compared to the preceding results, Strauch and Hahnel (2018) confirmed that indirect restoration worked better in patients with amelogenesis imperfecta. This is a disorder affecting tooth tissues, which produces “mottled and soft” enamel that leads to “problematic adhesive bonding to enamel surfaces,” and “impaired bond strength between enamel and resin” (Strauch and Hahnel 2018). Due to these circumstances, indirect restorations offer a higher probability of success and longevity than direct restorations (Strauch and Hahnel 2018).

Considering the results of the different application fields, direct and indirect restoration can give comparable results as soon as there is sufficient tooth structure (which means a maximum surface loss of 1–3 walls (Suksaphar et al. 2017) and healthy tissue (Strauch and Hahnel 2018)) and correct processing is ensured. Regarding efficiency, there was a tendency toward direct restorations for small defects. They were more efficient in processing than indirect restorations due to a less time‐consuming (Grivas et al. 2014; da Veiga et al. 2016; Azeem and Sureshbabu 2018) and simpler technique (Grivas et al. 2014; Vetromilla et al. 2020). Direct restorations were cost‐effective because of this (Chadwick et al. 2001; Grivas et al. 2014; Ahmed and Murbay 2016; Mesko et al. 2016; Afrashtehfar, Emami, et al. 2017; da Veiga et al. 2016; Vetromilla et al. 2020; Hardan et al. 2022), in addition to no additional lab costs (Grivas et al. 2014; da Veiga et al. 2016) and lower‐costing materials compared to indirect restorations made of porcelain or gold alloys (Grivas et al. 2014). Ahmed and Murbay (2016) concluded that direct composite restorations provided a cost‐effective alternative to conventional interventions, and (Vetromilla et al. 2020) pointed out that in daily practice, higher costs should be considered when choosing the material for restorations.

Using the AMSTAR 2 checklist, 40% of the included systematic reviews were rated with critically low confidence in the results, 25% were rated low, 20% were rated moderate, and only 15% were rated with high confidence (Table 4). These results can be attributed to the following AMSTAR 2 items, considered “critical flaw[s]” (Shea et al. 2017), which were particularly often not addressed. For example, 75% of the articles that were rated with critically low confidence in the results did not report their review methods or provide a list of excluded articles (Item 7), and half of these articles did not discuss the risk of bias in the results (Item 13). More than a third of the critically low rated articles did not use a detailed search strategy (Item 4), or technique for assessing risk of bias (Item 9), and none of these reviews, assuming a meta‐analysis had been conducted, investigated publication bias (Item 15).

Five articles had a low confidence mainly because they did not provide a list of the articles they had excluded (Item 7), or they did not investigate publication bias (Item 15) (Grivas et al. 2014; Afrashtehfar, Emami, et al. 2017; Vetromilla et al. 2020; Hardan et al. 2022; Kamposiora et al. 2023). Four articles passed only one critical domain, which gave them a rating of moderate confidence in the results (Ahmed and Murbay 2016; da Veiga et al. 2016; Suksaphar et al. 2017; McGrath and Bonsor 2022). There were three systematic reviews that were of high confidence in the results by presenting and discussing all the critical domains of the AMSTAR 2 (Shea et al. 2017) (Items 2, 4, 7, 9, 11, 13, and 15) to eliminate possible bias (Chadwick et al. 2001; Sequeira‐Byron et al. 2015; Afrashtehfar, Ahmadi, et al. 2017). These three systematic reviews presented the following results: Chadwick et al. (2001) concluded that amalgam showed the highest longevity compared to other direct filling materials and a better durability compared to indirect restorations in Class II cavities. Sequeira‐Byron et al. (2015) compared conventional direct restorations to single crowns on root‐filled teeth. The authors pointed out that there is insufficient evidence for one restoration type, as there was only one study with a high risk of bias included. Afrashtehfar, Ahmadi, et al. (2017) found that the greater the amount of remaining tooth structure, the better the treatment outcome in posterior root‐filled teeth. Indirect restorations in the form of post‐retained crowns give the best results.

### 4.3 Limitations and Strengths

This umbrella review had limitations. Only systematic reviews of clinical studies published in English were included; therefore, other potentially relevant systematic reviews published in languages other than English were excluded. Furthermore, the heterogeneity in the materials and procedures of primary studies included in the systematic reviews made an explicit comparison between both restoration types difficult.

However, our umbrella review also has many strengths. We provided a comprehensive search of systematic reviews in three major databases, and our search was likely representative of the literature published in English. Some evidence suggests that summary treatment effects of meta‐analyses including languages other than English do not produce major differences from those meta‐analyses limited to the English language (Morrison et al. 2012). We also followed the items reported by the AMSTAR‐2 checklist in conducting our umbrella review to provide a methodologically robust study.

## 5 Conclusion

In summary, the reports did not provide evidence to support a clear statement of whether direct or indirect restoration shows better results. Indirect restoration is more promising under certain circumstances, such as less tooth tissue, qualitative changes in tooth substance, and poor processing conditions. Regarding cost and processing efficiency, there is a preference for direct restorations for minor defects. Our research suggests that systematic reviews of high methodological quality are needed for scientific evaluations of failure in direct and indirect restoration.

## Author Contributions

**Clovis Mariano Faggion Jr:** conceptualization, data selection and extraction, formal analysis, writing original draft, supervision. **Mona Kimmel:** data selection and extraction, writing review, and editing.

## Conflicts of Interest

The authors declare no conflicts of interest.

## Supporting information

SupMat.

## Data Availability

Data available on request from the authors.
