# Supplementary material for: Systematic Reviews Comparing Direct and Indirect Restorations: An Umbrella Review That Examines Restoration Type and Confidence in Results
Source: Clin Exp Dent Res. 2025 May 26;11(3):e70149. doi: 10.1002/cre2.70149 (PMC12104983; doi:10.1002/cre2.70149)
Supplement: Supplementary file 1 — SupMat. [file CRE2-11-e70149-s001.docx]

**Supplementary files**

*Table S1. Search strategy*

**PubMed** #1 Search: (dental restoration[Title/Abstract]) OR (dental filling[Title/Abstract]) OR (dental inlay[Title/Abstract]) OR (dental onlay[Title/Abstract]) OR (tooth crown[Title/Abstract]) OR (dental crown[Title/Abstract]) OR (tooth crowns[Title/Abstract]) OR (dental crowns[Title/Abstract]) OR (dental porcelain[Title/Abstract]) OR (dental porcelains[Title/Abstract]) OR (dental amalgam[Title/Abstract]) OR (composite resin[Title/Abstract]) OR (composite resins[Title/Abstract])

#2 Search: (direct restoration[Title/Abstract]) OR (direct restorations[Title/Abstract]) OR (indirect restoration[Title/Abstract]) OR (indirect restorations[Title/Abstract])

#3 Search: #1 OR #2

#4 Search: (systematic review[Title]) OR (meta-analysis[Title]) OR (metaanalysis[Title]) OR (systematic literature review[Title])

#5 Search: #3 AND #4

**Web of Science**  #1 Search: AB=((dental restoration) OR (dental filling) OR (dental inlay) OR (dental onlay) OR (tooth crown) OR (dental crown) OR (tooth crowns) OR (dental crowns) OR (dental porcelain) OR (dental porcelains) OR (dental amalgam) OR (composite resin) OR (composite resins)) and English (Languages) and Review Article (Document Types) and Dentistry Oral Surgery Medicine (Web of Science Categories)

#2 Search: AB=((direct restoration) OR (direct restorations) OR (indirect restoration) OR (indirect restorations)) and Review Article (Document Types) and Dentistry Oral Surgery Medicine (Web of Science Categories) and English (Languages)

#3 Search: #2 OR #1

#4 Search: TI=((systematic review) OR (meta-analysis) OR (metaanalysis) OR (systematic literature review)) and Review Article (Document Types) and Dentistry Oral Surgery Medicine (Web of Science Categories) and English (Languages)

#5 Search: #3 AND #4

**Cochrane**  #1 Search: ((direct restoration) OR (direct restorations) OR (indirect restoration) OR (indirect restorations)):ti,ab,kw (Word variations have been searched)

#2 Search: ((dental restoration) OR (dental filling) OR (dental inlay) OR (dental onlay) OR (tooth crown) OR (dental crown) OR (tooth crowns) OR (dental crowns) OR (dental porcelain) OR (dental porcelains) OR (dental amalgam) OR (composite resin) OR (composite resins)):ti,ab,kw

#3 Search: #1 OR #2

*Table S2. List of excluded studies after full-text assessment, with reasons for exclusion*

| Author, Publication Year | Reason for exclusion |
| --- | --- |
| Almutairi et al, 2023 | Contradictory statements |
| Cribari et al, 2023 | Not about direct and indirect restoration |
| Naik et al, 2022 | Not about direct and indirect restoration |
| AlShehri et al, 2022 | Subject does not fit the topic |
| Lin et al, 2022 | Studies included are not performed on human adults |
| Gevert et al, 2022 | Studies included are not performed on human adults |
| Neto et al, 2022 | Not about direct and indirect restoration |
| Ozcan et al, 2021 | Other type of review |
| Fan et al, 2021 | Not about direct and indirect restoration |
| Arbildo-Vega et al, 2020 | Not about direct and indirect restoration |
| Xue et al, 2020 | Studies included are not performed on human adults |
| Jaiswal et al, 2020 | Studies included are not performed on human adults |
| Kozma et al, 2019 | Other type of review |
| Morimoto et al, 2016 | Not about direct and indirect restoration |
| Magno et al, 2016 | Not about direct and indirect restoration |
| Dawson et al, 2015 | Not about direct and indirect restoration |
| Moraschini et al, 2015 | Not about direct and indirect restoration |
| Yengopal et al, 2011 | Studies included are not performed on human adults |
| Manhart et al, 2004 | Other type of review |
| Burke et al, 1994 | Other type of review |
| Pendrys et al, | Not about direct and indirect restoration |
| Gauthier et al, 2021 | Full text not available |
